# Mimush Sheep and the Spectre of Inbreeding: Historical Background for Festetics’s Organic and Genetic Laws Four Decades Before Mendel’s Experiments in Peas

**DOI:** 10.1007/s10739-022-09678-5

**Published:** 2022-06-07

**Authors:** Péter Poczai, Jorge A. Santiago-Blay, Jiří Sekerák, István Bariska, Attila T. Szabó

**Affiliations:** 1 Finnish Museum of Natural History, University of Helsinki, PO Box 7, 00014 Helsinki, Finland; 2 Institute of Advanced Studies Kőszeg (iASK), PO Box 4, Kőszeg, 9731 Hungary; 3 Museomics Research Group, Department of Biosciences, Viikki Plant Science Centre (ViPS), University of Helsinki, PO Box 65, 00014 Helsinki, Finland; 4 Department of Paleobiology, National Museum of Natural History, Washington, DC 20560 USA; 5 The Pennsylvania State University, 1031 Edgecomb Avenue, York, PA 17403 USA; 6 Department of the History of Biological Science, The Moravian Museum, Zelny trh 6, 659 37 Brno, Czech Republic; 7 Vas County Archives Kőszeg, Hungarian National Archives, Kőszeg, Jurisics tér 2, 9730 Hungary; 8 BioDatLab, Balatonfüred, Bartók Béla u. 13, 8230 Hungary

**Keywords:** Animal breeding, Consanguinity, Genetic prehistory, Hereditary disease, Inheritance capacity, Scientific development

## Abstract

The upheavals of late eighteenth century Europe encouraged people to demand greater liberties, including the freedom to explore the natural world, individually or as part of investigative associations. The Moravian Agricultural and Natural Science Society, organized by Christian Carl André, was one such group of keen practitioners of theoretical and applied scientific disciplines. Headquartered in the “Moravian Manchester” Brünn (nowadays Brno), the centre of the textile industry, society members debated the improvement of sheep wool to fulfil the needs of the Habsburg armies fighting in the Napoleonic Wars. Wool, as the raw material of soldiers' clothing, could influence the war’s outcome. During the early nineteenth century, wool united politics, economics, and science in Brno, where breeders and natural scientists investigated the possibilities of increasing wool production. They regularly discussed how “climate” or “seed” characteristics influenced wool quality and quantity. Breeders and academics put their knowledge into immediate practice to create sheep with better wool traits through consanguineous matching of animals and artificial selection. This apparent disregard for the incest taboo, however, was viewed as violating natural laws and cultural norms. The debate intensified between 1817 and 1820, when a Hungarian veteran soldier, sheep breeder, and self-taught natural scientist, Imre (Emmerich) Festetics, displayed his inbred Mimush sheep, which yielded wool extremely well suited for the fabrication of light but strong garments. Members of the Society questioned whether such “bastard sheep” would be prone to climatic degeneration, should be regarded as freaks of nature, or could be explained by natural laws. The exploration of inbreeding in sheep began to be distilled into hereditary principles that culminated in 1819 with Festetics’s “laws of organic functions” and “genetic laws of nature,” four decades before Gregor Johann Mendel’s seminal work on heredity in peas.

## Introduction

In the second half of the eighteenth century, heredity was not yet defined as referring to the transmission of biological traits from one generation to another through the process of reproduction. Contrary to what is generally assumed of the origin of the laws of heredity—often coupled with the experiments of Gregor Johann Mendel (1822–1884)—interest in studying the transmission of traits and their theoretical conceptualization began to emerge in the eighteenth century. There is a growing consensus that the concepts of biological heredity were gradually constructed from the knowledge scattered in different domains, such as philosophy, jurisprudence, medicine, horticulture, and animal breeding (López-Beltrán 2006; Lidwell-Durnin 2020; McLaughlin 2007, p. 281; Poczai and Santiago-Blay 2022). Thus, the formation of the epistemic space of heredity as a scientific discipline required assimilating ideas from several other disciplines. As Steffan Müller-Wille and Hans-Jörg Rheinberger (2007) have shown, the phenomena of heredity did not go unnoticed in the eighteenth century; hereditary diseases (*haereditarii morbi*) and the passing on of physical monstrosities and mental peculiarities were linked to certain families (2007, p. 5).^1^ By the early nineteenth century, medical doctors were recording cases of insanity across Europe, pointing to heredity or “seed” as the most important factor behind madness. Theodore Porter has recently argued that physicians became obsessed with identifying insanity within family genealogy to prevent marriages that might have malignant outcomes (2018, pp. 19–58).

**Fig. 1:**
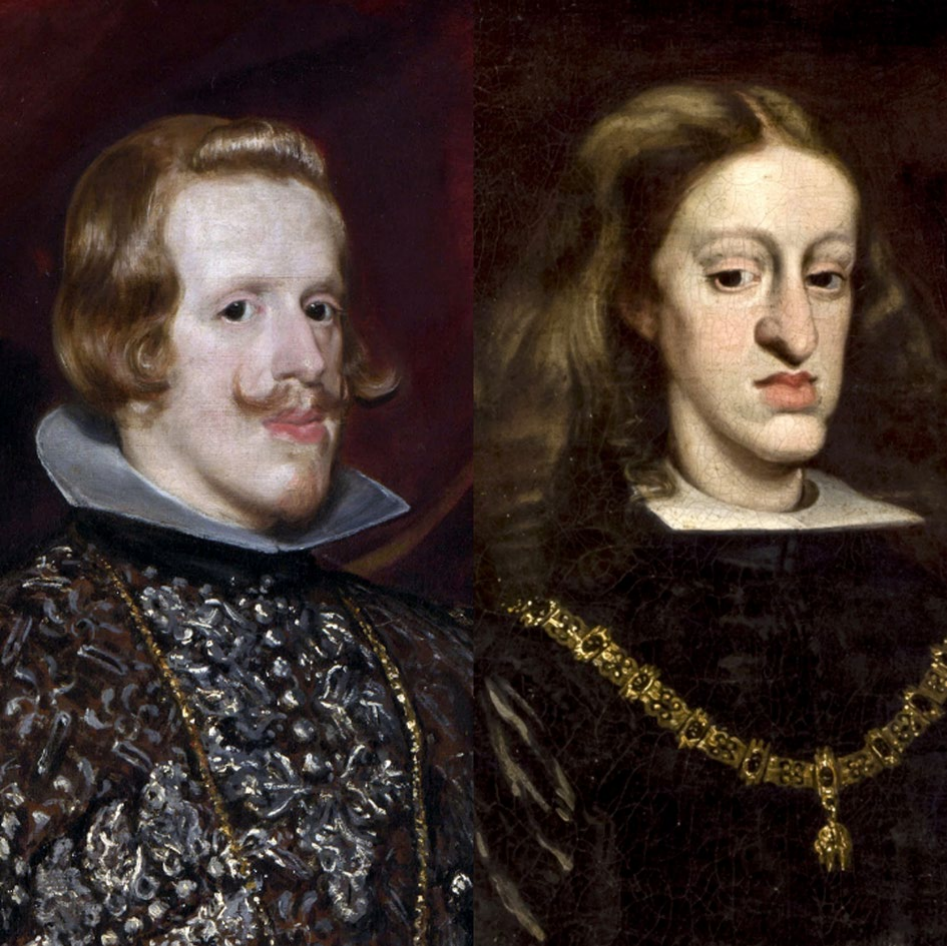
Portrait of Philip IV (1605–1665) on the left, and his son and successor Charles II of Spain (1665–1700) on the right. Oil painting of Philip IV in brown and silver by Diego Velázquez (1599–1660) ca. 1631 (National Gallery, London, UK); portrait of Charles II by Juan Carreño de Miranda (1612–1685) ca. 1685. (Images courtesy of the *Kunsthistorisches Museum*, Vienna, Austria)

In addition, the transmission of hereditary diseases (for example, epilepsy, apoplexy, and mania) was well known, given that many aristocratic families were associated with certain maladies. For example, gout was believed to be inherited along with royal titles and other “degenerative” illnesses, such as tuberculosis, colic, and madness (Wilson 2007, pp. 133–154). Consanguineous marriages were routinely contracted by European royal dynasties during the early modern period in order to establish political alliances (Ceballos and Álvarez 2013). Hereditary transmission of diseases and unusual physical features, such as mandibular prognathism, were well known in the Habsburg dynasty (the House of Austria) (Fig. 1). This facial characteristic, known as the “Habsburg jaw,” was apparent in at least nine successive generations of the family, very likely the result of inbreeding (Hodge 1977).^2^ As a result of consanguineous marriages, Charles II (1665–1700), also known as “The Bewitched” (*El Hechizado*), was so physically disfigured that the male line of the Spanish Habsburgs was extinguished (see Álvarez et al. 2009; Álvarez and Ceballos 2015). Based on such examples, it has become accepted that the biological understanding of heredity emerged partially from the observations of human diseases passed on from one generation to another.

As Christine Lehleiter noted, once observations of hereditary diseases had led to basic concepts of biological heredity, animal and plant breeders became major contributors of further explanations about heredity (2014, pp. 26–84). Since the livelihood of breeders was strongly dependent on the creation of better crops or domestic animals, they monitored the transmission of advantageous traits and crossed such animals in the attempt to create high yielding varieties with desired traits (Pawley 2018, p. 48). Farmers and breeders performed such applied experiments long before the emergence of the modern science of genetics. Sven Ova Hansson suggests that such attempts have been largely overlooked by scholars, since “action-guiding” experiments reflect a practical purpose—for example, producing wool with better elasticity (2019, p. 2). Basic research, on the other hand, aims to provide information on the actual mechanisms involved—cause and effect relationships—which serves an explanatory or other epistemic purpose (Hansson 2015, pp. 81–110). The two types of experiments (i.e., practical guidance and epistemic) are complementary and cannot replace each other, even if several parts of a directly action-guiding experiment are related to central topics of science.

This concept certainly applies to the empirical experiments carried out by the Moravian Agricultural and Natural Science Society (MAS, generally known as *Ackerbaugesellschaft*) in Brno (Brünn).^3^ During the Napoleonic Wars (1803–1815), members of MAS recognized that through inbreeding they could construct animals with better wool quality and quantity on a short time scale. Although inbreeding was rejected on religious grounds by Central European society, the Habsburg “Double Emperor” (*Doppelkaiser*) Francis II (1768–1835), nicknamed “the Good,” supported all activities to produce food and fabric essential for the army. This patronage by Francis II created the social niche and institutions that supplied the financial resources for MAS members to promote their investigations. During their annual meetings between 1815 and 1821, this society, administered by Christian Carl André (1763–1831), attempted to define a framework of generalizations and explanations that underpinned the new procedures for sheep breeding. Seeking answers to practical problems, they began to ask basic questions about heredity, probing a subject about which very little was known at the time (Orel and Wood 1998, p. 79). By summarizing the results of a series of ongoing breeding experiments, they set up a chain reaction for the growth of knowledge about biological inheritance among members of this private learned society.

The patronage of the absolutist Habsburg Emperor aided the cognitive legitimization of this new scientific endeavor by providing forums for the social acceptance of inbreeding, which improved the epistemological validity of the method. The rise, consolidation, and further growth of the physical sciences' prominence over several centuries had been made possible by similar patronage (Westman 1980, pp. 105–147). For astronomers like Nicolaus Copernicus (1473–1543), Tycho Brahe (1546–1601), Johannes Kepler (1671–1630), and Galileo Galilei (1564–1642), the patronage system and royal standards had formed a new agenda in their search for the true system of the world (Jardine 1998, p. 49). Mario Biagioli revealed how Galileo used the patronage of the Medicis to become an influential academic in the culture of absolutism (1993, p. 59). This all turned to dust when he lost the support of his two most important patrons during the events of what is now called the Galileo affair (Westfall 1985, pp. 11–30; Finocchiaro 1989). Astronomers promised that the information they created would not only advance the understanding of the world but also the reputation and power of their patrons. The members of MAS did not simply turn breeding experiments into a body of timeless theory on heredity; they also produced an extensive amount of cheap wool for the military campaigns of the emperor, who was struggling to keep his power.

Scholars have speculated about the reason for the liberal André’s departure from Brno in 1821 and whether it might have been because of his opposition to the absolutist policies of the Habsburg Empire (Wood and Orel 2001, pp. 234–250). In this paper, we suggest, rather, that André’s resignation as an influential academic of MAS was due to the controversy surrounding the debate over consanguinity. In what is known as the “inbreeding debates,” members of MAS deliberated about the scientific bases of inbreeding to gain better control over the method. They came to the conclusion that consanguineous matching permanently alters the interior structure of organisms and that it followed certain genetic laws (*die genetischen Gesetze der Natur*), as coined by Imre (Emmerich) Festetics [‘feʃtetɪtʃ] (1764–1847) from Kőszeg, Hungary.^4^ His Mimush [mɪmʊʃ] sheep, which was created through inbreeding, provoked a debate about whether the method produced abnormalities against natural law.

After the end of the Napoleonic Wars (1815), the pressing military need for wool ceased, and Francis II introduced strict censorship, which created a conservative reactionary environment less tolerant of social controversies (Sked 2008; Goldstein 2010; Emerson 2013; Bachleitner 2017). Based on the careful examination of contemporary documents, newspapers, and censorship reports written by the political police (*Polizeihofstelle des Innern*) led by Klemens Wenzel Nepomuk Lothar, Prince von Metternich-Winneburg zu Beilstein (hereafter Metternich) (1773–1859), often called “The Coachman of Europe,” we suggest that critical examination of inbreeding and the controversy surrounding consanguinity played a pivotal role in the changing leadership of the MAS.^5^ André’s editorial activities surrounding his *National Calendar* and the discussion of incestuous mating led to malicious public anecdotes about aristocratic families and the emperor, which eventually led to André’s downfall. His departure to Stuttgart left a huge void in the cultivation of knowledge in Moravia and the scientific life of Brno. For over two decades, members of the Brno society struggled to overcome the obstacle that has become known as “the spectre of inbreeding” (*das Gespenst der Verwandtschaftszucht*) (Nestler 1837a, p. 273; d’Elvert 1870, p. 183).

Herein, we ask, what impact did this obstacle have on early efforts to understand heredity? We propose that pre-Mendelian debates about heredity should be investigated from a different perspective. We hope that our work will provide a more complete historical background for the Brno scene and explain in detail the unfavourable circumstances scientists had to face in nineteenth century Central Europe. Our work also provides a detailed explanation about the intellectual milieu of the private learned society of MAS, of which later Mendel also became a member. Lastly, we characterize the atmosphere of inquiry created by this society, which provided a fitting prelude for Mendel’s later work.

## The Enigma of Inbreeding

Debates about sheep breeding, with scholarly deliberations on the question of heredity, were closely intertwined with philosophical and political questions in Brno during the early nineteenth century. During the Napoleonic Wars, there was a scarcity of fine wool obtained from Merinos, or “noble sheep” capable of producing the best wool, which had previously been imported from Spain (Jones 2016, pp. 24–56). Members of private learned societies in Central Europe—mostly factory owners, philanthropic aristocrats, intellectuals, animal breeders, and natural scientists—developed an interest in wool improvement. Working together, they wanted to produce large amounts of fine wool in a short period of time to satisfy the needs of the armies fighting on the battlefields. During the early nineteenth century, several factors contributed to strengthen the interest of governments, manufacturers, and agriculturalists in the question of wool (Mattone and Simbula 2011). The explosion of the fashion for woollen cloths, tapestries, and fabrics led to a demand for good quality wool throughout Europe (Fontana et al. 2016, pp. 275–304; Gayot 2002, pp. 633–666). This resulted in a rich sequence of studies on the wool industry and agronomic literature, with naturalists as protagonists (Sanna 2011, p. 714). Thus, it became urgent to establish a society that was specifically devoted to sheep breeding (André 1812, p. 183). In 1814, thanks to the personal support of Francis II, the Sheep Breeders’ Society (SBS) (*Schafzüchtervereinigung*) was founded (Festetics 1815a, b). In his speech, André praised the emperor and expressed his gratitude for his generous patronage (1815, pp. 93–111).^6^ Consequently, the city of Brno, also called the “Moravian Manchester,” became a major industrial centre for wool production in the socially and ethnically diverse Habsburg Empire (Freudenberger 1977, p. 17).

Central Europeans looked to the methods of plant and animal breeding in England, where agriculture had led to scientific progress (Lerner 1992, p. 12). Breeders of the British Isles aligned with the machine age to produce popular new breeds of crops and farm animals. The New Leicester sheep, bred by Robert Bakewell (1725–1795), was an example of how enlightened breeding could transform sheep into “machines, for converting herbage, and other food for animals, into money” (Sinclair, 1832. p. 83). Bakewell perfected sheep breeding, increased animal growth rate, and maximized the proportions of useful tissue based on minimum food intake. His success was grounded in excellent methodological approach: he pursued inbreeding (“breeding in-and-in”) in a closed stock, which helped him decide that “seed” had a more important role than climate in shaping the animal’s body. Bakewellian refinement quickly gained popularity, although consanguineous mating was rejected on religious grounds in the European continent. André began to explore “the enigma of inbreeding” with scientific thoroughness, publishing reviews about the method and its applications (André 1800, p. 47). These papers, published from 1800 to 1805 in his journal *Patriotisches Tageblatt* (PTB, Patriotic Daily News), stressed the advantages of inbreeding and attempted to change the minds of the breeders and the public to accept consanguineous matching of sheep.

For those arguing against inbreeding, degeneration of sheep was a practical confirmation against human incest, which thus gained biological endorsement for the legal term *bastardy*. The confluence of poverty, immorality, and gender was evident in contemporary interpretations of the moral character of bastard-bearers. In English society, these cultural tendencies influenced the moralistic assault on the reproductive habits of the poor and led to reforms in family law (Cody 2000, p. 133). In German lands, incestuous relationships were legally prohibited. To explain moral objections against consanguineous marriages, the Prussian lawyer and reformer Gottlieb Carl Svarez (1746–1798) used examples from sheep breeding in his lectures (Svarez 1960, p. 317). As Lehleiter has suggested, the acceptance of inbreeding required the separation of religious, social, and legal questions from biological phenomena, which was a step many were not yet willing to take (2014, p. 51). The appearance of inbred “sheep bastards” (*Schafbastarde*) were understood as degeneration, and such “freaks of nature” were not allowed to have a lasting influence on nature’s overall design (Poczai and Santiago-Blay 2021).^7^ Central European society was unwilling to accept the artificial modification of sheep, since they had adopted them as religious symbols reflecting their understanding of how redemption was achieved (Harney 2004, p. 3, Fig. 2; Orel 1997, pp. 315–330).

**Fig. 2:**
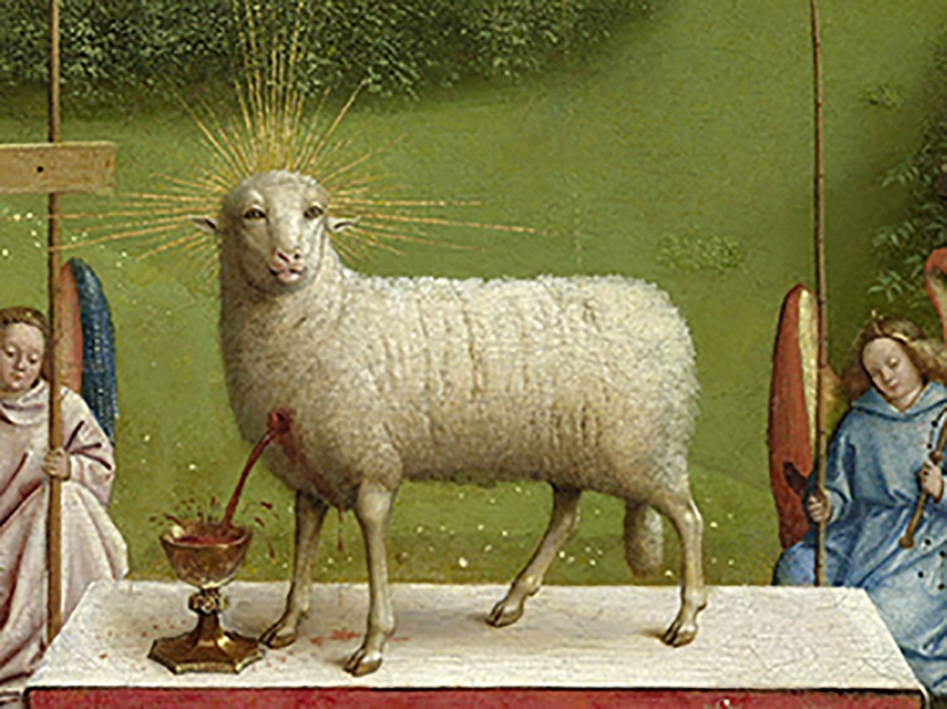
The Ghent Altarpiece: The Adoration of the Mystic Lamb (*detail*). *Altarpiece* by brothers Hubert and Jan Van Eyck from the collection of Sint-Baafskathedraal Gent, Belgium. (*Photo* by Dominique Provost)

Consanguineous pairing was opposed by notable animal breeders and scientists, including Franz Fuß (1745–1805), professor of agriculture at the University of Prague, who rejected inbreeding due to the potential harmful effects of genetic degeneration of the progeny (Fuß 1795). Christian Baumann (1739–1803), a Cistercian monk, suggested changing the mating of rams every three years to avoid degeneration (Baumann 1785, 1803). Johann Georg Stumpf (1750–1798) from Saxony concurred with such statements, pointing to the *Histoire Naturelle* of Georges-Louis Leclerc de Buffon (1707–1788), who supported crossbreeding as a model for improving farm animals (Stumpf 1785; de Buffon 1760, p. 152). According to Buffon, breeders could use the forces of nature (*moule intérieur*) in moulding animals to their purposes, but inbreeding should be avoided (de Buffon 1753, pp. 215–217). Only a handful of progressive-minded breeders rejected incestuous taboos in the Habsburg territories: Ferdinand Geisslern (1751–1824) of Hoštice, known as the "Moravian Bakewell," and Count Imre Festetics of Kőszeg, known as the “Hungarian Geisslern” (Anon. 1818a, p. 329). They were the main Central European breeders of the early nineteenth century who verifiably used inbreeding to improve the quality of wool.

André also joined the escalating debate, advocating inbreeding in hopes of purging the Buffonian spirit from the minds of breeders.^8^ The progressive Johann Petersburg (1757–1839), manager of the sheep breeding farm of the Archbishop of Olomouc (Olmütz), following in the footsteps of Geisslern and Festetics, was outraged by Buffon’s views on inbreeding. Petersburg circulated a pamphlet, later published by André in 1804, wherein he laid out his views against Buffon and asked Fuß to provide solid evidence of the harmful effects of inbreeding.^9^ The Austrian Bernhard Petri (1767–1853) also urged breeders to overcome their prejudice of inbreeding as a precondition for success. He was convinced that the Spanish created a genetically fixed race (*genetisch befestige Rasse*) of Merinos by making accidental variations endure (*zufällige Varietäten bleibend zu machen*) in the progeny using selective inbreeding without talking or writing about it (Irtep 1812, p. 10). The German translation (1804) of George Culley’s book *Observations on Livestock* prompted André to publish an article about the inbreeding methods of Bakewell. According to a footnote, the original article was to be published in the volumes of PTB in 1805, but publication was interrupted by the sharp reactions of the imperial censorship (André 1809).^10^ In high-quality wool production, harsh disputes between the agricultural world and politics were not isolated phenomena. Sanna has shown that social structures of the feudal system were the main obstacle preventing the modernisation of sheep breeding in Sardinia (2011, pp. 705–734).

Conservative policies dramatically affected both the work of breeders and natural history education in Central Europe.^11^ The political police attempted to intercept “dangerous thoughts and nip these in the bud,” while a special unit (*Oberste Polizei und Zenzurhofstelle – Highest Police and Censorship Court*) maintained censorship over the writings of academics and, more generally, over the entire press (Doyle 1978, p. 67). Foreign books on inbreeding fell under harsh censorship, narrowing the options of those who wished to inform others about this method. The publication of PTB was suspended, and André had to establish a new journal entitled *Economic News and Treatises* (*Oekonomische Neuigkeiten und Verhandlungen* or *ONV*).^12^ André reacted to the suspension of PTB by planning to leave the Habsburg Empire and to resign as secretary of MAS. “The emperor himself kept me back,” he said in a letter to his friend Professor Julius Franz Borgias Schneller (1777–1833), and “Count Lažanský, the chancellor requested me in a very flattering official letter to resume my pen” (Münch 1834, p. 335).^13^ André made two conditions to continue his work: first, a more liberal censorship of his writings; second, free admission of all books sent from abroad. This was allowed and afterwards confirmed by an official decree, issued and signed by the emperor in 1805, which eased the imperial censorship against André’s writings (Novotný 2002, p. 618). Count Prokop Lažanský (1771–1823), the enlightened regional chancellor, also protected André until his resignation.

This incident illustrates how important it was for the emperor to ensure a supply of wool for his armies. In 1805, Napoleon declared himself emperor of the French, and Britain with its allies, including the last Holy Roman Emperor Francis II, formed the Third Coalition against the French Empire. In this situation, nothing was more important than ensuring supplies for the soldiers, which heavily depended on wool production. André’s expertise and sheep-inbreeding were pivotal in reaching these goals. André thus continued his work with a large-scale plan supported by Count Hugo Salm (1776–1836), establishing a greater institution in Brno to study several branches of natural sciences, including animal and plant breeding.^14^ To mitigate any further concern, André and Salm named the new institute after their patron, Franzens-Museum (now the Moravian Museum). It was established on 19 July 1817 (Kroupa, 1998, p. 173).^15^

## Mimush and the Innermost Secrets of Nature

In this extremely unfavourable environment, the association organized a series of meetings between 1816 and 1821, which were intended to settle decades of dispute and controversy surrounding the effects of consanguinity (*Blutsverwandtschaft*) and inbreeding (*Inzucht*). Rudolf André (1792–1825) observed that when inbreeding is applied, something perpetually unique (*constant originelles*) develops in the offspring, which is kept in the organization of sheep and passed down through generations derived entirely and solely from pure blood relations (*aus lauter Blutsverwandten hergeleitete*) (André 1816, pp. 6–7). In response, his father André formulated fifty urgent questions for society members, including: What makes races different? Do internal organic structures (seed) induce constancy in a race, or do external conditions (climate) produce changes that can be fixed by breeding? He anticipated that these questions must be solved “before we can come closer to the truth,” since “here we are penetrating the innermost secrets of Nature (*die innersten Geheimnisse der Natur*)” (André 1818a, b, p. 303).^16^André hoped that through the “triumph of cash,” breeders would reveal the scientific truth on how the quality and quantity of wool could be increased through inbreeding. He initially tried to steer the debate on a strict scientific basis but failed to explain the theoretical principles of consanguineous mating. He focused on how the shape and traits of animals can be changed through generations by introducing the phrase “artificial selection” (*künstliche Zuchtwahl*) (André 1812, pp. 181–183).^17^ He also concluded that artificial selection coupled with inbreeding were powerful methods of improvement that could lead to an entirely new theory of generation.

“Sparks and flames erupt[ed]” at the meeting in mid-May 1817, when Imre Festetics displayed his sheep, called Mimush, which “developed a special shape” (Festetics 1819a, p. 11; Anon. 1819, p. 176; Anon. (K.) 1858, p. 310).^18^ Through systematic inbreeding and 15 years of long-term selection, Festetics had “concentrated” valuable characteristics (for example, straight wool, silver shine, low fat, thickness, wool density and length) in Mimush (Festetics 1819b, pp. 19–20; 1822, p. 730). The traits of this closed race (*abgeschlossen Rasse*) were incredibly well suited for the fabrication of light but strong materials (Festetics 1818, p. 552). Mimush was hastily ridiculed and criticized by the prominent Austrian breeder Baron J. M. Ehrenfels (1752–1843), who stated that such “refined bastard sheep arising from consanguinity are indisputably harmful in breeding” (Anon. 1818b, p. 298; Ehrenfels 1817, p. 91; Bartosságh 1837, p. 307). Upon Ehrenfels’s request, a separate committee inspected six rams from the Mimush line for signs of “organic debilitation” (*organische Schwächung*) (Anon. 1818c, p. 90).

According to Ehrenfels, the essence of animal organization was found in the sky, soil, and food of the homeland, which were responsible for “climatically building up the perfect shape of the animals.” These were then “echoed in their descendants” (Ehrenfels 1817, p. 91). In other words, constancy in inheritance was a direct effect of the “climate.” He explained that “features of the original descent” (*Merkmale der originalen Abstammung*) imposed by nature were irreversible and unalterable. “Nature seems to have indicated the borderline of wool quality,” and it would be “fruitless counteraction to work against Nature” because “it punishes those who undertake her daring” (Barteinstein 1818, p. 82). Ehrenfels stressed that unconditional inbreeding was harmful for two reasons: 1) it was against ancient principles (*Grundsatz der Alten*), which prohibited reproduction within family lines; and 2) for natural historical reasons, since “nature wants to work according to the principles established by herself” (Ehrenfels 1817, p. 91). He repeated that inbreeding was against the “main plasma of animal organization,” and, to avoid “bastard-like reversions” (*bastardartige Rückschläge*) and “natural climatic degeneration,” consanguineous matching should not be practiced in sheep breeding (Anon. 1818b, p. 298).^19^ Christian Carl André added that unconditional inbreeding practiced through several generations could result in organic debilitation (André 1818a; b, p. 303). As to consanguinity, he insisted that consequences should follow some kind of “physiological natural laws [or: law of nature]” (*natürlichen physiologischen Gesetzen*), therefore, stronger theoretical bases needed to be established to explain the changes observed through inbreeding (Bartenstein 1818, p. 81; André 1818a; b, pp. 298–303).

## Genetic Laws and Organic Functions

Although opinions about inbreeding differed, Ehrenfels reserved the possibility that he was mistaken, and he stated that he would accept the explanations if the nature of inbreeding was revealed on theoretical and practical grounds (Ehrenfels 1817, p. 94). André thanked the chancellor and the police for their lenient censorship and called upon Festetics to summarize his views in response to Ehrenfels (André 1818b, p. 308). Detailed reports of this debate were published alongside Festetics’s papers in the extraordinary supplement of *ONV*. Given the sensitivity of the subject, one report was signed by an anonymous participant identified as “Doctor of Philosophy” accompanied by André’s editorial notes (Anon. 1818b, c; André 1818b), while a separate report was also written by the residing president of the association, Baron Emanuel Bartenstein (1769–1838) (see Bartenstein 1818). Festetics admitted that the points listed by Ehrenfels could be true from a “purely physiological (*rein physiologisch*) point of view,” but to “illuminate the meaning of his system” he formulated his theoretical explanation under five “fundamental laws” (*Grundgesetze*) about “organic functions” (*organische Funktionen*) related to inbreeding:

1. I associate organic weakness … with the following definition: the subject in an otherwise good state of health is unable to perform and maintain its organizational functions by/through natural laws or a relatively long period of time.
2. I include among the organic functions everything that the laws of nature obviously require from the subject to preserve its self-organization (*Erhaltung seiner selbst*) and to propagate it in subjects resembling itself.
3. Robust constitution is related to the preservation of self-organization, which is partly inborn and which may partly increase or decrease by upbringing.
4. Precisely this robust constitution is necessary for the emergence of healthy entities resembling their ancestors in the process of reproduction. Healthy fathers often produce less appropriate offspring. Thus, the constitution, regardless of the state of health may weaken.
5. If traits that I desired for my purposes are fixed in the constitution of the Mather and the Father and variation appear in the offspring, these are either freaks of nature (*Spiele der Natur*) or the ancestors were not adequately equipped with the required traits. (Festetics 1819a, pp. 9–10)

Festetics tried to answer the question of whether any subject arising from consanguinity agrees with natural law or “lies outside of nature’s bounds.” Does consanguinity prevent physical entities from integrating their organic functions? By this, he meant the conservation of self and reproduction of offspring resembling their ancestors. He specified that growth and development of entities depend on environmental responses, which together with inborn components alter the structure and composition of entities. Stable inner conditions, or a “robust constitution” as Festetics called it, is required for the entities to reproduce heathy progeny, which can deteriorate regardless of their state of health. But what if both parents exhibited healthy constitutions and had been carefully selected to possess the desired traits? His answer was that even in these cases variation (*Änderung*) could appear in the progeny, which he called “freaks of nature” or “aberrations.” In his last sentence, he also maintained the possibility that parents may not possess the desired traits sufficiently enough to transmit it to their offspring. (In a footnote, André added that there must be an error in the transcript of this part of the sentence.) Festetics admitted that these explanations are not exhaustive, because “here we are only striving to search for the truth” and the contradicting issues are only “verified by pure experience” (Festetics 1819b, p. 19).

In the final chapter of his paper entitled “About Inbreeding” (*Ueber Inzucht*), Imre Festetics (1819c) investigated whether this method had a harmful effect on generation by breaking down the transmission of traits through degeneration, or whether it led to the contrary state—more certain heredity? In a practical consideration, does inbreeding debilitate sheep to a certain state, where they cannot mate and lose their “noble” characteristics, or is it the exact opposite, that they yield better, more refined wool? According to the “humble opinion” of Festetics, the following four paragraphs contain the “Genetic Laws of Nature”:

- Animals of healthy and robust constitution plant and transmit their characteristic traits.
- Traits of the predecessors, which are different from those of their descendants, appear again in future generations.
- The animals which have possessed the same acquired traits through many generations can have divergent characters. These are variants, freaks of nature, unsuitable for propagation when the aim is the heredity of desired traits.
- Scrupulous selection of stock animals is the most important precondition for the successful application of inbreeding. Only those animals possessing the desired traits in abundant amount, can be of great value for inbreeding. (Festetics 1819c, p. 169)

In the first law, Festetics linked heredity with health and a robust constitution. André, in a footnote added to the term “scrupulous selection,” specified: “In my opinion, this underlines the main point.” Existence was tough for a breed imported into a strange environment, as pointed out by Ehrenfels. Degeneration was an ever-present danger. “Noble” blood would not be transmitted readily by a sick male, nor could desirable lambs be expected from a weak female. In the second of his laws, Festetics assured his fellow members that when a character skipped a generation it need not be considered a sign of degeneration. Such gaps in heredity were commonly observed and offered no barrier to eventual breeding success. The changes he referred to in the third law were of a different nature: deviations from normality which had to be excluded from the blood stock. Such aberrations might arise from a variety of causes, possibly connected with deviations from health and fitness as referred to in the first law and noted in the fifth point about organic functions (Festetics 1819a, pp. 9–10). The fourth and most significant law referred to mating among chosen bloodstock from which aberrations had been expelled. To inbreed in such circumstances, each trait being separately considered, was the way to maintain high quality (Szabó 1991).

Festetics believed his “organic and genetic laws” to be evidence of daily natural phenomena underpinning fundamental functional processes of “natural history,” which proved that the method of inbreeding could not be defined as “manipulation against ennoblement” that “goes against natural law” as stated by Ehrenfels. How could Nature act against herself? Festetics verified his laws based on his observations and experiences (*theoria cum praxi*) in sheep, horse, goat, swine, horned cattle (*Magyar Szürke*), and poultry breeding (Festetics 1819a, p. 10; 1822, p. 729). He asked, How many homogeneities and heterogeneities does nature have? Is it just a fantasy to ask if the same analogy of natural law could occur in the plant kingdom? Among plants, Festetics saw that “climate” has a more detrimental effect and that one must work with tireless efforts to understand what the rules imposed by nature are to itself (Festetics 1819c, p. 170). In his publication, Rudolf André verified Festetics’s observation and added that only “animals from homogenous animal breeds” (*homogene Racethiere*) possess the necessary “organic strength” to produce the “noble race” and that “consanguineous mating is the only available means by which the propagation of valuable traits in a pure state can be achieved in the progeny” (André 1819a; b, p. 161). He also repeated his previous expression that “such animals possess the natural capacity and the potential (*Anlagen*) to reach higher perfection assisting Nature” (André 1816, pp. 94–96).^20^

**Fig. 3:**
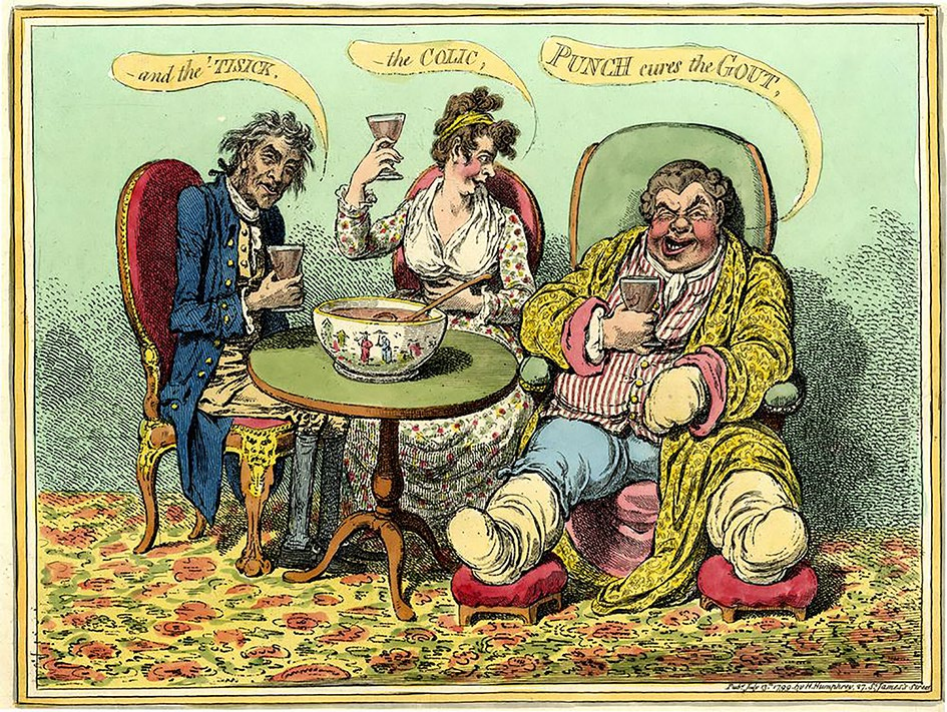
A caricature of the “hereditary diseases” of the aristocracy. The hand-coloured etching entitled “Punch cures the Gout, -the Colic,- and the ‘Tisick (1799),” created by the famous caricaturist and printmaker James Gillray (1756–1815), also called “the father of political cartoon,” humorously illustrates the “noble characters” of the aristocracy. (Print provided courtesy of The British Museum, London)

The debate swiftly changed course after Christian Carl André noted that, although such “heterogeneous and homogenous nuances” might be hard to notice among plants, it is certainly easy to observe them among humans: “blue-eyed blonds exhibit weaker constitution when several generations marry in the closest possible relationship” (André 1819a; b, p. 26; Fig. 3). These were dangerous statements, even in a harmless natural historical and animal breeding debate, from a person who had previously been characterized with a “French friendly attitude” by Metternich’s political police.^21^ The question remains, noted André, of “how will the position of Nature work in the case of our civilization?” Are the similar regularities observed among animals “*eo ipso* detrimental for the full health of the organism,” or does inbreeding have deviating influences in humans (André 1819a, b, p. 26)? Festetics’s opinion was that inbreeding does not produce debilitation in humans (Festetics 1819c, p. 170). He did, however, observe “characteristic facial features, manner and behaviour” among different communities living in small, depopulated areas of Hungary where people intermarried among each other (Festetics 1819a, p. 10).^22^ In his final statement, he turned to André and asked whether human inbreeding could be scrupulously observed to validate his “genetic laws” (Festetics 1819c, p. 169). André, in response, pointed as an example to aristocratic and royal families who practiced consanguineous marriages:

There are princes and other families, where this closely observed bond indeed expressed a striking *air de famille* [family resemblance], but not directly to the advantage of the descendants, whereon debilitation cannot be overlooked. Perhaps this example explains my meaning better than a long deduction. (André 1819a; b, p. 26)^23^

These statements had a life-changing impact on André’s life and, through it, on the entire long-term functioning of the private learned society of Brno.

## The *National Calendar* and Doctor Eisenbarth

A sign of the new and lively interest in hereditary discourses in Brno was echoed in the pages of *ONV*, which appeared in two volumes (vols. 19 and 20) and supplements in 1820. The debates on wool improvement continued after Ehrenfels accepted Festetics’s explanations about inbreeding. In the subsequent meeting, Festetics suggested that “scientifically aiding manufacturers and giving sheep breeders a clear set of rules” would clear the “scientific society of prejudices, arming it with the science of wool perfection” (Festetics 1820a, p. 115). Festetics suggested that the association should determine which “characteristics the perfect wool should have,” then “name these traits accurately and scientifically” by “mathematical definition and ordering wool grades” and traits such as smoothness, elasticity, and resilience (Festetics 1820b, p. 26). Moreover, Festetics (1820a, p. 115) emphasized that the following two questions were of utmost importance. The first involved whether or not one might be able to find these characteristics in their highest form *in combination* in nature; the second was, if these characteristics were not found together in their highest level, is it really necessary to differentiate between wool characteristics?

Festetics advised the members of the society to use “mathematically ordered characteristics” and to trace them in crossbreeding where “experience must show if the new trait of the wool can be kept constantly and predominantly in the future herd” (Festetics 1820a, p. 116). For the characteristics of the wool to be properly enumerated and traced in later generations, the members of the association set up a breeding register in which the characteristics and ancestry of the animals were recorded. The breeders were asked to preserve a small wool sample from their animals and to attach these samples to special sample cards.^24^ To evaluate wool grades, Rudolf André created a micrometer device and demonstrated his scheme for characterizing sheep breeds.^25^ Festetics believed that this “will be a milestone in the science of breeding” (Salm 1820, pp. 33–34; Festetics 1820a, p. 27).

While the members of the association appeared to have solved the “innermost secrets of nature,” society in general continued to nurture prejudices about the method. Following the debates, rumors, and anecdotes took wing that were associated with biblical taboos (Anon. 1821a, pp. 615–616; Anon. 1821b, pp. 357–360; Anon. 1821c, pp. 551–552; Anon. 1821d, p. 483; Anon. 1843, pp. 608–609).^26^ At their center was André, the secretary of the society, by which a negative shadow was cast on the activities of the association (see Kallbrunner 1925, pp. 111–112). According to these leading anecdotes supposedly spread by his ill-wishers, unremitting in their hostility, André insulted aristocratic classes and the estates of the royal realm by spreading falsehoods and deception about their *air de famille* (Anon. 1821a, pp. 615–616; Anon. 1821b, pp. 357–360; Anon. 1821c, p. 551).^27^

**Fig. 4:**
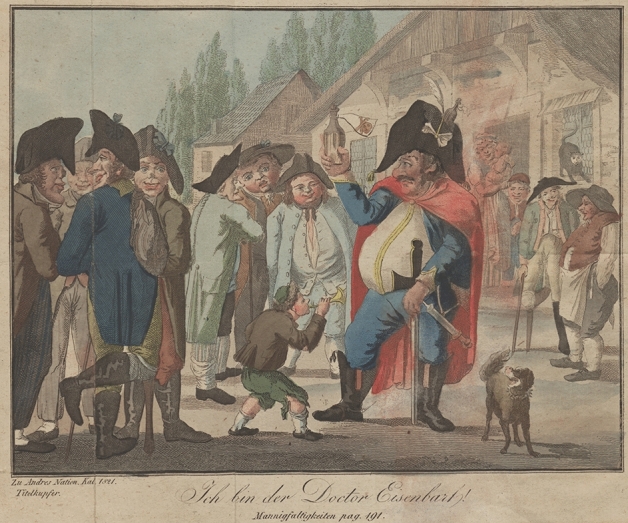
The fold-out image of Doctor Eisenbarth from Christian Carl André’s *National Calendar* (1821). (Courtesy of the Department for Manuscripts and Rare Books, Austrian National Library [104894-B.1821])

André also published a *National Calendar* from 1810, which sold in large numbers (Kucher 2007, p. 39). It also gave him the opportunity to influence the culture of the middle class and farmers, in addition to the more educated subscribers of *Hesperus* and *ONV*. André’s intention was to initiate a change through education and to create “a popular sense, a political, artistic, technical intelligence, from which all the great effects which we find in history prevailed” (Anon. 1821b, pp. 357–360).^28^ It was in the Emperor’s *National Calendar* intended for the year 1821, printed on 20 August 1820, that André published a detailed genealogy of the European aristocratic and royal families—including Metternich, the head of the political police—revealing their intermarriages (André 1821, pp. 1–152). In addition, the opening page of the calendar included a fold-out colour print that displayed a caricature of Doctor Eisenbarth, a boastful doctor who was said to have killed many patients with his “miraculous cures” (Fig. 4).^29^ The fabled barber surgeon, also well-known from a popular mocking song about “curing people his way,” was depicted surrounded by soldiers possessing the “Habsburg jaw.” Rumours spread that André had ridiculed the “noble characteristics of the Habsburg emperor.”^30^ Considering the ongoing debates about consanguinity in MAS on the noble character, the “Habsburg jaw” of the emperor appeared to be portrayed as a debilitating feature that publicly questioned his capabilities in commanding the army. The Eisenbarth figure falling under imperial censorship illustrates how the concept of heredity in nineteenth century Brno played a powerful role in debates over debilitation, monstrosities, morality, race, classes, social change, education, and politics.

Together with the aligned rumors of inbreeding, André’s *National Calendar* attracted the attention of the censors. According to the *Literarisches Conversations-Blatt* (*Conversation Magazine*), the decree of confiscation arrived together with the calendar to post offices and distributors.^31^ The publication was banned and destroyed; however, limited copies were later sold after removal of the censored Eisenbarth figure and pages of unwanted content.^32^ André surrendered his duties in Brno by resigning as the leading scientific figure of MAS and SBS. He was followed by Hugo Salm, who resigned as the chair of MAS to emphasize his disagreement with the accusations against André (Anon. 1821a, p. 615). In fact, André called 1820 “the red year in the journal’s existence” and assured authors of *ONV* and *Hesperus* that their submissions and contributions were not lost and would be published later (André 1820, pp. 209–212).^33^ Several members supported André, while Hugo Salm formulated a petition in which he praised André’s merits and asked the members of the SBS to support such a petition.^34^ The members of MAS applied for an award, a golden medal, for André at the regional office in Brno, to signify their respect for what he had accomplished and to encourage him to carry on. As a testimony to André’s merits, the SBS sent a delegation consisting of Count Colloredo, Count Festetics, and Baron Barteinsein to the regional chancellor’s office (Anon. 1821a, p. 615).^35^ Festetics testified in favour of André that “he has been inspired to visit the Olympus of Agriculture, which widened his knowledge and he became more enlightened.”^36^

The suppression of André, “the soul of everything in Brno,” shocked the scientific community in other private learned societies, and the process against him was viewed as an act of harsh censorship. Police reports dated between 1819 and 1820 considered André’s various connections with foreign private learned societies to be suspicious, especially his connection to the Polytechnic Society of the Bavarian Kingdom and his honorary memberships issued by other, similar societies.^37^ In the eyes of the Viennese government, André had become an “antimonarchist Jacobian Conspirator.” Moreover, in their view, his connections with intellectuals from private learned societies indicated the influence of revolutionaries. Nevertheless, no praise or action on behalf of this committed teacher, writer, economic adviser, organizer, however justified, was enough to prevent the axe from finally falling (Wood and Orel 2001, p. 250). At the very least, the subsequent development of natural history, as well as further discussions about heredity and inbreeding, were slowed down. Festetics wrote to Salm that his heart was broken and that he “feels that everything that they have been working on for the last two decades is now lost.”^38^

## The Spectre of Inbreeding

André’s physical separation, and the negative stigma associated with inbreeding, created a credibility gap among the members of the society in terms of continuing inbreeding-related investigations of “the innermost secrets of nature.” Nearly three decades later, this obstacle became known among members of the society as “the spectre of inbreeding” (Nestler 1837, p. 273; d’Elvert 1870, p. 183). Sheep breeders, scientists, and society itself were all divided. One camp—previously led by André, Festetics, and Salm—was supporting inbreeding to create “noble” lines of sheep to “concentrate” valuable characteristics. On the other hand, there were those who feared the unpredictability of, and the danger of disastrous consequences arising from, consanguinity, particularly in terms of fertility.^39^ This controversy, heightened by religious considerations about transgressing God-given law, was now also hallmarked by stories of the *National Calendar* associated with André’s departure. André’s special licenses, issued in 1805 after the inbreeding-article incident in PTB, resulting in an elevation on his level of censorship, were withdrawn in the “red year” 1820 in reaction to the content of his *National Calendar*. According to the police reports, André was forced to leave Brno in September of 1821, subsequently becoming *persona non grata* to the Monarchy.^40^ André wrote the following on leaving Brno:

After the beginning of this year, my licenses were suddenly withdrawn, and I was urged to respect the law. So, I have no other choice but to choose one of the many countries where more sensible laws are in place. Besides Rome, Austria is probably the only country where literary treasures are seized, and this wealth is deprived of me. Elsewhere the circulation of books is generally free, and individual works are not prohibited, packages are not piled up, opened or retained. (Münch 1834, p. 335)

He moved to the cloth-manufacturing city of Stuttgart and became an adviser to the King of Prussia, later the Emperor of Germany William I (1797–1888).

After the great 1816–1817 famine in Württemberg, the king strove for the improvement of agriculture, with the help of novel scientific knowledge, to prevent such catastrophes from happening again. As an acknowledged agricultural-development expert who encouraged the practical application of natural sciences, André was assured citizenship of the Kingdom of Württemberg, as well as enough financial support to carry out his activities (Voigt 1831, p. 638). André held nearly the same offices in Stuttgart as he had in Brno. He became the secretary of the Württemberg Agricultural Society (WAS) and published a periodical *News Magazine of the Royal Württemberg Agricultural Society in Stuttgart* (*Correspondenzblatt des Königlich Württembergischen Landwirthschaftlichen Vereins Stuttgart*), which was similar to his *ONV*, edited in Brno. Settled in Stuttgart, André continued openly defending inbreeding, selection, and individually controlled matings (*Sprung aus der Hand*) in his new journal (André, 1823a, p. 100; 1823b, p. 278; 1824, p. 388). His efforts applying natural sciences in practice were supported by the initiator of the German empirical-rational agricultural teaching, Johann Nepomuk Hubert von Schwerz (1759–1844). Schwerz founded an Agricultural Institute in Hohenheim (today the University of Hohenheim) and without prejudice supported André’s efforts applying inbreeding in Württemberg (Schwerz 1822, pp. 316, 333). André influenced the young WAS member August von Weckherlin (1794–1868), who concluded that heredity is the foundation of all breeding methods and that through inbreeding constant laws of inheritance can be determined (Weckherlin 1825, 1846, 1860).

With André’s exile, Moravia lost a pioneer of the Industrial and Agricultural Scientific Revolution. Shortly before his departure for Stuttgart, André invited Georg Carl Ludwig Hempel (1770–?), the secretary of the Pomological Association of Altenburg, Germany, to Brno. There, Hempel gave a lecture about the use of artificial plant pollination in creating new varieties of domesticated plants through hybridization. André was persuaded that animal breeding would offer a template for plant breeding; therefore, he devised a proposal for the Pomological and Oenological Society (hereafter Pomological Society) to be formed as a new branch of MAS, which was chaired by Jan Sedláček von Harkenfeld (1760–1827). André also chose his successors: Professor Johann Karl Nestler (1783–1841)—who was in personal contact with André through correspondence—was intended to continue lecturing about animal breeding, while Franz Diebl (1770–1859) was supposed to carry on work related to plant breeding as a curator in the newly opened Franzens-Museum (Orel 1975, p. 234). André also published Hempel’s paper in the “red year;” the paper reviewed the work of Thomas Andrew Knight (1759–1838) on outbreeding (see Campbell 1983).^41^ Hempel suggested that hybridization could be an ideal method to identify the desired characters in breeding and stressed that understanding the “laws of hybridization” is the key to achieving this (Hempel 1817, 1820). As his last act in Brno, André wanted to set the stage for further long-term investigation of the transmission of traits from one generation to another.

Without André, it was impossible to shift the balance in support of inbreeding, and members of the society called upon Festetics to once again explain consanguinity in detail. He reported his observations and his application of inbreeding in poultry farming, where he concluded that inbreeding can be practiced in any animal species (Festetics 1822, p. 729; Teindl 1822). He stressed that inbreeding “imperatively follows nature’s breeding rules” because “nature punishes other deviant behaviour.” He reported that, in the case of some desired traits, which were selected through careful breeding, one trait “must be the pair of desirable characteristics that complement each other” when they are transmitted further in breeding livestock. Festetics also observed that “specific traits of homogenous animals are sometimes added to the qualities of the stock” without further changes.^42^ However, he failed to provide further explanation for these observations and was clearly puzzled about the nature of the phenomena. Nonetheless, this did not make him insecure about his previous laws, nor the application of inbreeding. In his closing statement, he assured his fellow members for the last time that inbreeding was not harmful (Festetics 1822, p. 731).

Festetics no longer spoke of inbreeding or genetic laws, at least according to our current knowledge. After 1821, almost all members of the society started to follow the principle of *audi, vide, tace—*hear, see, be silent. Meanwhile, André tried to continue his work in Brno through his sons Rudolf and Emil, but the political police prevented this. In response, André—now in Stuttgart—issued honorary memberships and diplomas to previous MAS members in Moravia, which was closely monitored by the Austrian state authorities.^43^ The letters sent to André by the sheep breeders were first sent to Salm in Rájec-Jestřebí. Salm then sent these letters to Stuttgart by way of the publisher Johann Friedrich Cotta (1764–1832) to escape the attention of the police.^44^ The ploy did not work, however; letters were intercepted, and communication with André became almost impossible. André lost his son Rudolf to pneumonia in 1825, isolating him even more from Brno. André asked for help from the members of MAS and volunteers to continue his editorial work and influence on scientific development in Moravia (André 1826, pp. 165–168). He was also desperately looking for a new publisher and further connections in the Kingdom of Illyria to print his works and other unpublished material sent to him by members of MAS.^45^ André wanted to deliver these manuscripts to Moravia, but, a few years later (1831), he passed away, and some manuscripts were lost and therefore remained unpublished.^46^ According to Albrecht Thaer (1752–1828), the Sheep Breeders’ Society was dead in 1823 (Anon. 1823, p. 45).

In Stuttgart, André continued to publish the journal *Hesperus* until his death*,* in which he spoke openly about the censorship against his writings (André 1823a, b; c, p. 142; André 1831, p. 656). The embittered André could not refrain from pouring out his anger and mockery on the Austrian police, and he became “notorious” for attacking and criticizing the Austrian government in his papers (Kallbrunner 1925, p. 113).^47^

## The Great Mysterious Workshop of an Almighty Nature

By 1822, the development of animal and plant breeding in the Moravian Agricultural and Natural Science Society had stimulated the growth of knowledge in Brno, encouraging experiments and scientific disclosure about heredity. Later, members began to refer to the observed transmission of different traits with the terms “history of heredity” (*Vererbungsgeschichte*)” and “hereditics” or “the lore of heredity” (*örökléstan*) (Nestler 1837, p. 277; Haubner 1857, p. 461). Inbreeding, when carefully applied, made inheritance more certain, but the “great mysterious workshop of an Almighty Nature” (*die große geheimnißvolle Werkstätte einer allmächtigen Natur*)—as Bartenstein referred to the problem in 1836—remained unanswered, causing frustration among members of the society (Teindl et al. 1836, p. 304). In the decade following André’s departure, the scientific discussion of the crucial issue of inbreeding became anonymous, demonstrating the sensitive nature of the subject. One such anonymous article from 1824 discussed the constancy of traits in subsequent generations of animals:

Nature teaches from the blade of grass to the cedar, from the mite to the elephant, that in the superabundance of her products, imperfect products, imperfect formations never disappear completely… Happy are those, who do not see, and yet believe. (Anon. 1824, pp. 231–232)

In the void of nearly a decade after André’s removal, the support of Cyrill Franz Napp (1792–1867) helped the MAS begin to flourish again.^48^ As an abbot, patron, protector, and social superior, Napp attempted to fill the vacuum André had left in Brno. Napp, described by František Matouš Klácel (1808–1882) as a “scientist, secret freethinker and patriot,” had a broad interest in the growth of scientific knowledge in animal and plant breeding (Dvořáková 1976, p. 133).^49^ On 26 January 1826, the Episcopal Office forwarded the concerns of Francis II to the local governor’s council (*Gubernal Presidium*).^50^ According to the letter, the lecturing of agricultural and natural sciences was allowed to continue in Brno, with the conditions that teaching in “the propagation of organic bodies through procreation (*die Vermehrung organischer Körper durch Zeugung*) by means of two sexes, on the characteristics and selection” was significantly reduced.^51^ Napp, who was himself interested in the topic, defended the curriculum in a response dated 17 November 1826. The letter—signed by Count Mittrowsky—stressed the long-term goal of Abbot Napp to establish the teaching of artificial selection within the walls of the monastery, where “prudence will be carefully applied” about the subject. In these efforts to discover the truth about heredity, Napp became the most influential leader in MAS (van Dijk et al. 2018, pp. 351–352). He organized a plant nursery with 3,400 seedlings near the village of Šardice, which was later moved to the monastery in Brno, described by fellow MAS member Ferenc Schams (1780–1839) as “an institute created for practical experiments” (Schams 1836, pp. 5–18).

The first member to openly attach his name to writings about the “reproduction of organic bodies” was Bernhard Petri, who stated that tenderness of fine wool was dependent on the natural cohesion of inner parts of the body determined by the genetic force (*genetische Kraft*) (Petri 1826, p. 638). He also added that a new epoch of natural sciences had begun, where domestic animals and natural species were not the products of creation (Petri 1827, p. 4). Following Petri’s example, in 1829 Nestler published his own lectures about the influence of consanguineous mating on hereditary defects (*vererbbare Fehler*) (Nestler 1829, p. 378). Nestler was the first to openly defend inbreeding nearly a decade after André’s removal. His paper reflected on the empirical knowledge gained through artificial selection over the decades. Nestler explained phenomena through the lens of a broader natural historical perspective, giving numerous examples of animals, plants, and humans.^52^ At the end of his lecture, using the wool sample register, he attempted to evaluate mathematically how the overall characteristics of wool blend together over six generations of sheep (Gliboff 2013, pp. 27–44). Nestler also defined the “inheritance capacity” (*Vererbungsfähigkeit*) of sheep in terms of a “developmental history” (*Entwicklungsgeschichte*) linked to heredity (Nestler 1837, p. 277).^53^

Nestler brought more attention to animal breeding in his lectures and publications, while in the Pomological Society the effects of artificial selection on the survival of the offspring were discussed. Anton Schönberger from Mokrouše (Mokrausch) noted that “Nature drives and cultivates” plants, which “compete among themselves for existence,” and the repressed forest trees overgrown by the more successful ones die out (Schönberger 1825, p. 62). He suggested selecting only such prosperous individuals for plant varietal crosses. The president of the society, Jan Sedláček, saw the potential of mixing such characteristics via sexual reproduction, much as sheep breeders had done in the past (Sedláček 1826, p. 162).

Francis II granted permission to the Philosophical Institute of Brno to fill the position of a professor in 1827.^54^ Through Napp’s intervention, the position was given to Diebl (Orel 1975, pp. 232–236). As a professor, he pioneered theories on artificial selection, focusing more on the function of plant hybridization in breeding (Diebl 1835). Diebl thought that naturalists examine solely hereditary characteristics when classifying plants. He was aware that, although they may vanish under certain circumstances, these characteristics may reappear for reasons currently unexplained by natural history (Diebl 1839, pp. 270–271). Diebl emphasized that plant physiology, on the other hand, supports the idea that such characteristics emerge as a result of hybrid fertilization under certain circumstances (Diebl 1829, pp. 177–179). The majority of cultivated plants have subspecies; research indicated that their unique characteristics are not stable and are subject to change as a result of the causes listed above (Diebl 1830, p. 133). While natural historians prove the presence of variations, according to Diebl, the plant breeder conducts scientific research on plants with the aim of developing novel, more productive forms (Diebl 1829, pp. 177–179).

Napp, the *adsum* “Moravian André” in Brno, encouraged members to explain how nature creates new species of animals and plants via forces that are beyond the control of humans, and how breeders control the reproductive process and utilize changes through crossings. In the 77-year-old J. M. Ehrenfels, this fresh stimulus (begun by the young Napp) sparked a renewed interest in the theoretical foundation of breeding and natural history. Variability and constancy, he said, were two sides of the same coin, originating from the “genetic force, the mother of all living creatures” (Ehrenfels 1829, p. 130). In this regard, he saw a distinction between breed (*Rasse*)—which might be constant—and variety (*Varietät*)—which could change over time and through generation. Ehrenfels explained that such variation was the primary lever of nature in the formation of living beings in the lifeless chaos of matter (Ehrenfels 1831, p. 137). Following the ideology of Festetics, he referred to this process as “genetic mixing (*genetische Vermischung*),” referring to the combination of variable traits in constant races (Ehrenfels 1829, p. 137). He added that living beings cannot depart from the power of elements, since their upbuilding is bound by “genetic barriers (*genetische Schranken*)” (Ehrenfels 1829, p. 134). In his views races contained traits, which were the results of the climatic-genetic expression (*klimatisch-genetische Ausprägung der Organisation*) of the external and internal organization of organisms (Ehrenfels 1829, p. 139). Therefore, deviations might return by genetic means (*Abweichung auf genetischem Weg*) in mating generations, which could be utilized in the effective and faster transformation of forms in breeding (Ehrenfels 1829, p. 139).

Ehrenfels contrasted his ideas with the philosophy of Johann Gottfried Herder and Hippocrates as well as with the experiments of Lorenz Florenz Friedrich von Crell, Johann Friedrich Gmelin, William Harvey, Carl von Linné, Samuel Thomas von Sömmerring, Caspar Friedrich Wolff, and Albrecht von Haller (Ehrenfels 1829, 1831).^55^ He concluded that the interaction of “climate, nutrition and generation” through the “genetic force” have inflexible principles and rules in the arrangement of natural forms, and these mechanisms can be used in breeding through “genetic intervention (*genetische Einschreitung*)” (Ehrenfels 1829, p. 140). August Mayer (1802–1873) concurred with Ehrenfels, claiming that humans may harness the forces of Nature by "removing and adding something to modify shapes [of creatures] differently" in breeding, similarly to natural processes, via "genetic mixing" (Mayer 1831, p. 146). Petri agreed with Ehrenfels and Mayer, but he noted that such a new theory must be rooted in practice; otherwise, it is nothing more than a mere observation (Peteri 1833, p. 367). Contrary to Ehrenfels, Rudolph Ritter von Löwenfeld (1813–1853) questioned whether, through such mixing, entirely new strains (*Stamm*) of animals with novel traits could be created (Löwenfeld 1835). Nestler disagreed with Löwenfeld and reiterated the conventional concept of Nature, which acted to eradicate any deviations from transmitting their traits in the offspring (Nestler 1836, p. 181). Nestler was making a clear distinction between what occurs in breeding (*Entwicklungsgeschichte*) and what happens in nature (*Vererbungsgeschichte*) (Nestler 1837, p. 277).^56^ These ideas were also incorporated in a new natural history textbook by Nestler and Diebl (1836) intended for university students and fellow members.

## Heredity as a Research Project: What is Inherited and How?

Artificial selection, as coined by C. C. André, was thought to offer proof for the “genetic force” to resist environmental influence via its fundamental “genetic laws,” as Festetics envisioned. Members were still fumbling in the dark, speculating on the principles and laws behind the transmission of characteristics from one generation to the next (see Tiendl et al. 1836). The topic emerged again from the remark of Count Dominik Eugen Wrbna (1786–1848), a Vienna-based MAS member, who expressed the worry of many when he observed the irregularity of sheep characteristics in the offspring of crossings, a problem that required investigation. He emphasized the need to progeny test rams prior to their introduction into the reproductive process:

[Breeding rams] cannot be sold at any price if their advantages are inherited by its offspring, and if they are not inherited, then it is worth no more than the cost of wool, meat, and skin. (Barteinstein et al. 1837, p. 227)

Nestler noted that Wrbna had posed the question of heredity to the society and that it urgently needed to be investigated by all means of natural history, including anatomy and physiology (Nestler 1837, pp. 300–303). He noted that “the question of heredity is very difficult,” and again vehemently advocated the practice of inbreeding, which received a “crusade *in foro publico*” but could be pivotal in approaching the truth about heredity (Nestler 1837, p. 289). Ehrenfels agreed with Nestler, and added in response that sheep must have changed in their organic body parts through genetic influence (*durch genetische Einwirkung*) (Ehrenfels 1837, p. 2). He added that breeders understood how nature creates living forms and thus they set a new direction for science (Ehrenfels 1837, pp. 3–4). Reflecting on these comments, Abbot Napp drew attention to the fact that the topic had completely diverted from the original problem: “We are not dealing with the theory of the process of breeding, but the question should be: what is inherited and how (*Was wird vererbt und wie*)?” (Barteinstein et al. 1837, p. 227).^57^ The Hungarian Ede Bujanovics (1776–1855) emphasized that such investigation can only be carried out through the mutual interaction of natural and agricultural sciences combining empirical and theoretical knowledge (Bujanovics 1838, pp. 17–23).

Members of MAS sought to put the origin of differences and the transmission of similarities within a coherent theoretical framework, as expressed at their meetings and published in their newly established natural historical journal *Mittheilungen*. It was agreed during the Third Conference of German Agriculturists and Foresters in Potsdam (1839) to hold the following congress in Brno. The influential agriculturalist Alexander Lengerke (1802–1853) emphasized that the high level of innovative techniques in animal and plant breeding was a significant factor in selecting Brno as the site of the congress (Lengerke 1840, pp. 111–115). Some participants in the 1840 Brno Congress viewed the hybridization of plants as a random process. Napp defended the perspective of MAS, stating that “artificial fertilization is not a random affair,” but he admitted that the theory of this new breeding method was not yet known (Nestler 1841, pp. 337–339). Napp thus extended his previous questions regarding “what is inherited and how” with a third point: what is the role of chance in heredity? The issue he had so elegantly articulated could only be addressed via an entirely new experimental approach using fundamentally novel research methodologies. Ferdinand Stieber (1804–1885), a fellow MAS member and private docent in Olomouc who was inspired by Weckherlin from Württemberg, met Napp and subsequently Mendel. Stieber also added to the discussion that heredity—what he called *vis plastica*—acts mechanically and continuously, but the controversial method of inbreeding is a reliable way of fixing it in generations (Stieber 1842, p. 41; 1851, p. 123).^58^

Christian Carl André set the groundwork for information sharing through the press and meetings. His outstanding organizational abilities steered both the publication and meeting programs of the MAS in such a way that empirical knowledge grew gradually, which was beneficial to breeders. He succeeded in creating a spirit of cooperation in the society, which had a lasting impact. After the sudden death of Nestler in 1841, it was Napp and Diebl who gained the cooperation of fruit tree and crop breeders in developing new varieties by means of larger-scale hybridization. The manner in which Napp accepted the sheep breeder’s conclusion, that the laws of heredity could be defined only when inherited traits were considered separately from one another, has added significance in light of events. Their conclusion, reached on the basis of practical experience, deviated from the mainstream tradition of natural history. By the 1840s, the influx of cheap wool from Australia and other overseas territories, together with the death of famous breeders, had essentially bankrupted the industry, and the SBS ceased to exist for lack of economic interest. Napp, who remained interested in the study of heredity, recognised early on that the empirical regularities developed by sheep breeders without the “spectre of inbreeding” could be further investigated in plants (Napp and Diebl 1838).

In June 1843, the Brno St. Thomas Abbey sent a letter to the University of Olomouc to recommend that a suitable candidate continue his studies in natural sciences at the monastery. Professor Friedrich Franz (1783–1860), who taught physics and had previously lived in the monastery, recommended his student Johann Mendel to Abbot Napp. In his letter, Professor Franz stressed Mendel’s academic success and excellence in physics (Klein and Klein 2013, p. 185). In Brno, Mendel took up the friar’s name Gregor and found himself surrounded by likeminded priests whom Napp admitted similarly to the monastery.^59^ From his native village, Mendel brought with him to Brno an interest in growing plants. This interest immediately connected him with Diebl, whose courses in agriculture and natural sciences Mendel took for two semesters at the Brno Philosophical Institute, using the previously developed course books (for example, Nestler and Diebl 1836). Diebl's lectures were an ideal introduction for Mendel. Napp identified Mendel’s talent, provided the necessary education for his genius to blossom, and in 1851 sent Mendel to study at the University of Vienna. Upon Mendel’s return to Brno in 1854, Napp constructed a greenhouse in the monastery garden, which was further extended with an orangery (1855) and a hothouse (1856) for his experiments. Nothing could have been more natural for Napp, in the atmosphere of inquiry concerning heredity fostered by André, Festetics, and many others in his office, than to support the young Mendel's plant breeding studies. After all, wasn't it Napp who elegantly formulated the question "what is inherited and how?" However, the conservative atmosphere of MAS, grounded in empirical observations for practical applications, proved to be an obstacle for further scientific advancement.

Natural scientists in Brno tried to overcome this obstacle by establishing an autonomous Natural Science Society to advance pure science. Thus, in 1861, a conflict arose between the MAS and the members of the Natural Science Section (*Naturwissenschaftliche Sektion*) established in 1850, in which Mendel and Napp were members. Members of this section chose to conduct experimental investigations and test Nature's logic as a system (Sekerák 2006, p. 243). At the time, the notion of separate developmental stages of matter was the dominant paradigm.^60^ In the dynamics of a natural system, the components and their interactions began to take precedence over the elements themselves.^61^ Experimentation was considered critical by the members of the Natural Science Section. Their generalizations were targeted at mathematical and physical notions, compared to practical observation of the MAS (Šohajková 2000, pp. 9–19). They regarded mathematics and physics as precise disciplines, since they were founded on theoretical models. The teaching faculty at the Brno Technical College, where Mendel was a substitute teacher from 1854 on, provided the impetus for establishing an autonomous Natural Science Society (*Naturforschender Verein,* NSS) in 1861. Among the most prominent figures advocating for an independent society were Headmaster Josef Adolf Auspitz (1812–1889); Alexander Zawadski (1798–1866), a noted scholar, professor of physics, and Mendel's superior at Brno Technical College; Alexander Makowsky (1833–1908), professor of natural history and noted scholar who later collaborated with Mendel; and Gustav Niessl von Mayerdorf (1839–1919), an astronomer and botanist. The mayor of Brno, Christian d’Elvert (1803–1896), established the Society's headquarters in a new contemporary state school building in the center of the city. The place became the new home of associated members’ periodic meetings, where they reported on the results of their experiments. These reports were published in the *Proceedings of the Natural History Society in Brno* (*Verhandlungen des Naturforschenden Vereines in Brünn*).

At an 1864 meeting organized by NSS, horticulturists again discussed the role of inbreeding and bastardy. The then-72-year-old Napp, the only surviving member who had been present in Brno from 1810, had witnessed André's incident in the "red year," and had successfully reorganized the association, once again concluded that heredity "is much more a question of science than practice" (Orel 1975, p. 237). From this standpoint Napp again stressed that inbreeding is a theoretical problem. This was just the period when Mendel was finishing his paper to be presented to the NSS at the beginning of the next year. Courage and “prudence” applied among the monastery walls eventually broke through the cognitive paradigms and social prejudices about “the spectre of inbreeding.” Mendel displayed similar courage in his research. In the introduction of his 1865 lecture, he stated that “it requires a good deal of courage to undertake such a far-reaching task” to finally “arrive at a solution about the developmental history (*Entwicklungsgeschichte*) of organic forms” and to “establish their mutual numerical proportions in separate generations” (Mendel 1866, p. 4).^62^ In a letter to Carl Nägeli (1817–1891) dated 18. April, 1867, he further elaborated that under the current circumstances publication of one isolated experiment was doubly dangerous for the experimenter and also for the cause it represented (Stem and Sherwood 1966, p. 60).

Mendel knew that the results he obtained would not be compatible with contemporary scientific knowledge. Unfortunately, he was right; his audience could not comprehend his theory about particulate inheritance emerging from the intellectual background and many interactions between breeders and naturalists in Central Europe. However, Moravian agriculturalists immediately understood the epochal significance and practical meaning of Mendel’s experiments (Sekerák 2012, p. 182). Their assessment was the sole reaction he got in the nineteenth century; academic sciences only acknowledged his work 35 years later.

## Conclusion

At the 1839 meeting of the Moravian Agricultural and Natural Science Society, remembering the work of Imre Festetics, J. K. Nestler published a paper with the exact same title, “About Inbreeding” in which he asked: How did “the spectre of inbreeding” influence the early investigation of heredity? (Nestler 1839). Looking back, we can reflect on Nestler's question from several angles. We have to take into account the public—and society as a whole—that witnessed the André affair, the patronage system that operated under the absolutist rule and strongly influenced scientific work, and the functioning of institutions in Brno. The public, patronage, and institutional framework have all been demonstrated to influence the establishment and dissemination of certain research fields and methodologies. The André affair in various circumstances exemplify this concept.

Organic reproduction as heredity implies that the development of organisms is seen as a realm governed by structures or processes that extend beyond the monumental act of forming an individual being. In the early nineteenth century, experience proved that inbreeding increased the certainty of heredity: something consistently unique emerged in the organization of sheep and was passed on through generations of pure blood relatives. Breeders recognized that characteristics from the producer to the produced should correspond in both inner and outer organisation of living organisms. They had already put their understanding of racial transformation into practice, but the public was doubtful of the relevance and long-term effects of such alterations. The worry of a breed's deteriorating quality due to inbreeding also prompted many animal breeders to practice regular out-crossing. Many human communities have a repulsive aversion to incest, and this long-standing phobia is likely a consequence of this. Inbreeding was conceived to be innovative, but it needed to break through cognitive paradigms and social prejudices. The incest taboo was challenged as a cultural norm by supporters of Natural Law, although they were constrained by more conventional frameworks. Degenerations were defined as deviations from the original shape of an organism, and such abnormalities were not permitted to have a long-term impact on nature's general design. This prevailing ethical mindset has led to the erroneous assumption that there is a physiological prohibition against inbreeding in general. Deterioration is obviously made easier by inbreeding, but the few brave souls who have successfully inbred higher stock revealed that the path taken was only made feasible by the instances of degradation and are not its direct and consistent effect.

Not only did the issue of heredity become clearer to Central European breeders through inbreeding, but its rapid effectiveness was also recognized by Francis II, who supported the Moravian Agricultural and Natural Science Society financially and through special licences to its secretary Christian Carl André. His pragmatism was matched by that of André, although with different objectives. Francis II was a leading opponent of Napoleonic France and heavily relied on food and supplies produced in one of its major industrial centres in the “Moravian Manchester.” The emperor considered inbreeding an important means of consolidating his power, and he had every reason to do so, as the results of the breeders spoke for themselves. Breeders hoped that obtaining a deeper understanding of inbreeding would open novel possibilities of generating wealth and prestige by applying such basic knowledge in wool refinement. Under the influence of André, the debates of breeders from economic applications gradually shifted to natural historical explanations reflecting the favorable or unfavourable influence of inbreeding over the continuity of generations. André emphasized that heredity deserves to be the subject of a major physiological study that might lead to a discovery comparable to those made by Copernicus or Newton, continuing the path set by Maupertuis (André 1815, p. 103). André had shown an extraordinary ability to build a *modus vivendi* with the political regime that afforded MAS with the institutional and financial means necessary to undertake this scientific inquiry.

Westfall that such patronage provided no assurances the relationship between patron and client was constantly prone to dissolution since the client’s sole claim was the ability to further display the magnificence of the patron (Westfall 1985, p. 29). After the end of the Napoleonic Wars, the pressing military need for wool ceased together with the tolerance of Francis II. He introduced strict censorship, which affected André and members of MAS who, on the other hand, remained concerned about the scientific nature of inbreeding. André's academic influence in the *annus horribilis,* 1820, evaporated during the aftermath of the *National Calendar* incident, when he was held responsible by central censors for his liberal beliefs. After André’s death, efforts were made to suppress his memory; *Hesperus* could not publish a detailed obituary and D’Elvert (1870), in his detailed historical work about MAS, mentioned André with a certain embarrassment (Střesková 1966, p. 268). We can now formulate our answer: that this dispute was, at a minimum, a symbol of the restrictions that inhibited intellectual life in Brno and halted the development of early research on heredity as a scientific subject and debates on the method of inbreeding. The society was reorganized by C. F. Napp, an abbot who defended the matter of generation and reproduction, and, after nearly a decade of silence, under his patronage members recognized heredity as the central scientific problem beyond inbreeding. There are no records of Napp's probable participation in Mendel's pea experiments. Despite this, we can safely assume that he was aware that the issue he had defined so succinctly thirty years before was starting to be addressed by Mendel while he was protecting it.

Early investigators of “the lore of heredity” rejected the constancy of the race and elaborated a theoretical framework of individual potency, which treated heredity as a natural phenomenon influenced by the environment. They pondered on the mechanisms behind heredity and were unable to explain the “enigma of inbreeding” or the perplexing patterns of inheritance of different organisms. They left an indelible impact on many people on whom Mendel was destined to rely for advice and direction. No specifically established association had corroborated the existence of heredity more clearly, nor raised more doubts about it. Brno had been the hotspot of these debates just before Mendel entered the monastery. He must have benefitted from all of this at the time he began his experiments. According to Nestler, Napp had cast the seed of this question onto the proper soil, and the tireless work of pivotal MAS members such as Festetics, Diebl, and Ehrenfels created the “embryo” of a new epoch of natural sciences over several decades. This atmosphere of inquiry about heredity was perfectly suited to Mendel; thus, the seed of the “lore of heredity” gradually developed into “the luxuriant fruit of science.” It was among the circles of the Moravian Agricultural and Natural Science Society in Central Europe that heredity was recognised as a scientific problem, which was converted into a research question and later rose to an independent academic subject.

## Footnotes

1 It has been known since Hippocrates of Kos (c. 460–370 bce) and Aristotle (384–322 BCE) that certain diseases can be passed on from one generation to another, referred to as inherited diseases (Greek *Κληρονομικοί νόσοι* = *Klironomikoí nósoi*). The importance of hereditary diseases is also emphasized by the Transylvanian György Váradi Lencsés (1530–1593) in *Ars medica* (c. 1577) (Szabó 2013).

2 The “Habsburg jaw,” a physical characteristic, shared by many individuals of the family, was caused by the co-occurrence of maxillary deficiency (MD) and mandibular prognathism (MP); see the genetic study of Vilas et al. (2019).

3 The Moravian Agricultural and Natural Science Society (MAS), operating between 1806 and 1898, was a merger between the Society of Agriculture and Liberal Arts (*Gesellschaft des Ackerbaus und der freien Künste*) and the Society of United Friends for the Promotion of Natural and National History in Moravia (*Gesellschaft der vereinigten Freunde zur Beförderung der Natur- und Vaterlandskunde in Mähren*) initiated by André (Sekerák 2010, p. 199). The word “natural science” in the society’s name symbolises the new approach laid down by its members in the founding document, which aimed to study and understand nature as the “real” world (Kroupa 1998, p. 173; Sekerák 2012, p. 180).

4 Festetics’s genetics laws were first reported by Orel (1989) and Szabó and Pozsik (1989).

5 Documents numbered AT-OeStA/AVA Innere PHSt, also known as “*Brandakten*” (Fire Records) of the Austrian State Archives, Vienna were scorched and severely damaged in an accidental fire during the July Revolt of 1927 in Vienna (*Wiener Justizpalastbrand* – *Vienna Palace of Justice Fire*). Access to all these documents was granted by the Austrian State Archives based on an individual assessment of each page. We also examined a large volume of historical documents, contemporary newspapers, and archival letters that circulated among MAS members, held in the National Széchényi Library (OSZK), Budapest, Hungary and in the Moravian Archive (MZA) in Brno, Czechia. Our aim was to investigate these documents using real-time optical character recognition (OCR) implemented in Transkribus (Kahle et al. 2017, pp. 19–24) to uncover the motivation behind André’s resignation and to build a comprehensive historical background for the Brno scene (1780–1822) in relation to the inbreeding debates.

6 The full name of the society was the “Association of Friends, Experts and Supporters of Sheep Breeding for the achievement of an even higher and more thoroughgoing advancement of this branch of the economy and the manufacturing and commercial aspects of the wool industry that is based upon it” (*Verein der Freunde, Kenner und Beförderer der Schaftzucht, zur noch höheren, gründlichen Emporhebung dieses Oekonomie-Zweiges und der darauf gegründeten, wichtigen Wollindustrie in Fabrikation und Händel*). It functioned as an independent branch of the MAS. The SBS was the first animal breeding association established on the European continent, in replicating “The Society for the Improvement of British Wool” established in Edinburgh in 1791. Festetics (1815a) appealed for the foundation of a “Society for the Perfection of Sheep Breeding” (*Juhtenyésztést Tökéletesítő Társaság*) in Hungary, which was established ten months after SBS. Imre Festetics refers to SBS as the “Sheepy Bunch” (*Juhos Társaság*) (in Festetics 1815a).

7 The well-known physician Caleb Hillier Parry (1755–1822) stated that Bakwellian inbreeding is against Divine Law and reduces sheep to the state of eunuchs (Parry 1806, p. 471).

8 André also sent his son Rudolf to study at Geisslern’s farm. Rudolf later wrote a book about sheep breeding based on his experiences (1816). André’s younger son Emil, who later became the editor of ONV, took study trips related to animal breeding to Hungary. He died while on one of these trips in Kisbér (1869).

9 Petersburg even wrote a poem in defense of inbreeding, which was often cited in public circles. An accurate historical account of the circumstances is recorded in detail by d’Elvert (1870) II. pp. 145–152. André’s original printed work did not survive, but a description of its content can be found in K in Mähren (1811).

10 André wrote in the footnote: “This essay was still intended to be published in the *Patriotisches Tageblatt*. All readers may agree and wish that the incisive and knowledgeable author may continue his views and teachings.”

11 The titles of books that André and other MAS members borrowed from libraries or ordered from abroad were scrutinized by the authorities. The empire maintained special control over its education system through the political police (*Polizeihofstelle des Innern*) to ensure that “subversive” ideas would not infiltrate students and academics (Goldstein 2010, p. 74). Teachers were fired if they “demonstrated their incapacity to fill their offices,” or if they “misused their proper influence on the young, spreading harmful theories inimical to the public order and peace or destructive to existing political institutions” (McClelland 1980, pp. 219–220).

12 Details about suspending André’s journal are found in the police report AT-OeSTA/AVA Inneres PHSt Z 190 (Austrian State Archives) 1805. André’s new journal was also subjected to censorship before printing as stated in AT-OeSTA/AVA Inneres PHSt 1810-22b (fol. 35–43).

13 See also André (1818a, b, pp. 308–309) about the incident.

14 Seeking advice, they actively corresponded with Count Ferenc Széchényi (1754–1820), who had founded the National Library and the National Museum of Hungary in Pest-Buda (Budapest). The correspondence is found in the National Széchényi Library, OSZK Fol. Germ. 803 dated 10 October 1816. André understood the museum to be an instrument of progress and connected the institution with a set of ancient monuments, works of art and collections of natural specimens in scholarly cabinets. André’s museum was to serve people; it was to be a tool of an active attitude toward the present and toward the reality which surrounds people, and which improves them. The fundamental change in the importance, which André had implicitly in mind, was thus the shift from culture to civilization, which the museum was to serve.

15 The idea of the creation of the Moravian Museum dates to 1803, as initiated by André. The proposal to establish the institute was commissioned by the Moravian estates and drafted by the historian Josef Hormayr (1781–1848) while he was a guest in Hugo Salm’s castle, soon after his release from the “Prison of Nations” (Špielberg Castle 1816; Kroupa 1998, p. 173). In the introduction, Hormayr stressed the merits of Mittrovskýs and their contribution to Moravian cultural life. But to avoid Hormayr’s bad reputation after years spent as a political prisoner, the plan was presented as André’s own idea. Although Francis II accepted the establishment of the museum as part of the *Ackerbaugesellschaft* on 19 July 1817, its official foundation was announced by the Governor of Moravia in a decree only on 24 March 1818; the museum opened on 5 May 1820.

16 André stressed the importance of consistent terminology with reference to the work of Gottlieb Werner (1749–1832) who achieved a scientific breakthrough in mineralogy by standardizing scientific terminology (André 1818a). André pondered whether similar breakthroughs could be achieved in explaining inbreeding via clarifying terms.

17 The term *artificial selection* is reminiscent of the expression introduced much later by Charles Darwin (1809–1882) when he hit upon an analogy with natural selection.

18 The term *Mimush* originally referred to theatrical works that (like tragedy and comedy) trace their roots back to folk plays with or without cultic overtones and portrayed a typical scenario in the life of the ordinary people in relatively brief acts, often with a pretentious, sometimes obscene crudeness. A detailed description of the physical characteristics of Mimush (also spelled as Mimusch or Mimus), often called the “silk-sheep (*selyembirka*),” can be found in the reports: Bartosságh (1837, p. 307); Bartosságh (1823, pp. 58–59); Festetics (1819b, pp. 19–20); while the pedigree of Mimush is detailed in Festetics (1819a, pp. 11–12).

19 For a full account of the latter, he guided his readers to his previously published works and stated that it is well known that there is “no constant reproduction among bastards in nature” (Anon. 1818b, p. 298).

20 The term *Anlage* was widely used by MAS members in a metaphysical sense referring to a predisposition for transmitting certain traits to offspring. They advanced the concept by implying a difference between inner and outward organization, with the former dictating the latter. *Anlagen* were defined by Mendel as the determinants of distinct characteristics acquired through the germ cells.

21 André’s French friendly attitude (*franzosenfreundliche Haltung*) is detailed in the report of AT-OeStA/AVA Inneres PHSt 1823–9239 (Karton 1065), Fol 23 Austrian State Archives, Vienna. Further description of the personality of Rudolf and C. C. André by state authorities can be also found in AT-OeStA/AVA Inneres PHSt 1823–9239 (Karton 1065), Fol 23.

22 Festetics also mentioned that in the case of a so-called civilized person, the intellect has to be considered alongside physical traits since, in civilization, “scrupulous choices without prejudices, a great education system, and the traits of vivacity, health and mother love” are an important part of upbringing, which constantly alters degeneration. He was convinced that the “people living in civilization tied by business deals should get closer to a natural way of life, both in their households and treatment of their animals” (Festetics 1819c, p. 170).

23 During the eighteenth and nineteenth century, the lower classes believed that gout, colic, hysteria, or tuberculosis were inherited along with noble titles. Hereditary aristocracy was based on the concept of transmitting the "noble essence," which necessitated bloodlines free of interclass marriage. For example, in early nineteenth century France, doctors abandoned Galenic traditions in favour of counselling aristocratic families – a practice known as *hérédité* – on how to keep the essence of nobility in their marriages in order to prevent illnesses and deformities. The rapidly expanding masses of landless poor, believing themselves to be subjects of this hereditary system, criticized the aristocracy's "noble characteristics," and the ruling class saw this as potential dangers to social order (see Pomata 2007, pp. 145–152; López-Beltrán 2004; Cartron 2007, pp. 155–174; Wilson 2007).

24 In a meeting organized by the association in 1837, J.K. Nestler asked the members to search for patterns in the “hereditary history of different wool traits.” Breeders kept collecting the wool sample cards and stock registers for more than two decades without finding any correlation in the heredity of traits (see Nestler 1837).

25 Nestler also gives further details about the application of this device (Nestler 1836, pp. 183–187).

26 Note that the delicacy of the subject is well illustrated by the fact that all the newspaper articles reported on the incident appeared anonymously.

27 In a personal letter dated 20 November 1820, Professor Schneller wrote to André that these ill-wishers might be the Austrian medical doctor and writer Franz Sartori (1782–1832), who worked in the book revision office in Vienna and acted as an important editor to a rival calendar, and Josef Hormayr (see Münch 1834, p. 337; Anon. 1843, p. 608). André conducted a form of literary opposition against the Monarchy, while Sartori and Hormayr represented the opposite antagonistic pole. Though André and Hormayr were acquainted and had met after Hormayr was released from Spielberg, their relationship was inimical.

28 André’s motto on the title pages of the *Calendar* describes his intentions: “There are instructors of humanity who are not discoverers, but who are really significant and immortal individuals, those who take what others have found and make it known to others, even if it is well known in one country.” By contrast, Francis II intended to use the *Calendar* for making an “imprint on the poorest minds of hundreds of thousands and millions” similar to “commandments or religious catechisms” and “speaking about the highest consecration of power” written “in the language of the Holy Land and Apostles” to achieve “glory and celestial sparks for the enlightenment of the people” (Anon. 1821b, p. 358).

29 Johann Andreas Eisenbarth (1663–1727), otherwise known as “Doctor Eisenbarth” – although he was never officially awarded with the title of a medical doctor – was an oculist and barber-surgeon who travelled throughout Germany from 1685 until his death, with a large entourage of up to 120 people, including entertainers, harlequins, and musicians. After arriving at a location, he performed treatments such as amputations, bloodletting, pulling teeth on patients, often surrounded by large crowds, and accompanied with loud music. He was well known for his brutal methods, and many believed that he belonged behind bars. He was famous for his miracle cures and, thus, received numerous honors, titles, and awards from the aristocracy in response to his “medical treatments.” During 1800 a student drinking song entitled “My name is Doctor Eisenbarth” (*Ich bin der Doktor Eisenbarth*) became popular in mocking the memory of the doctor.

30 According to the surviving versions of the anecdote, André’s work discredited the entire army as well as the emperor, and the rumour has spread that “in the war of 1809 an entire battalion tore up in front of the clouds of dust stirred by a herd of oxen” (Anon. 1821c, p. 551; Anon. 1843, p. 608).

31 The *Literarisches Conversations-Blatt* (1820–1825), later entitled *Blätter für Literarische Unterhaltung* (1826–1898), was published in Leipzig by Friedrich Arnold Brockhaus (1772–1823). Brockhaus and his publishing house came under scrutiny and pressure from censors for his liberal political views. These tensions mounted during the Metternich era and involved a number of German states. For example, the editor Lorenz Oken (1779–1851), who was a professor of zoology in Jena, investigated the subject “if we really have press freedom” (*ob wir wirklich Pressefreiheit haben*) in his writings published by the Brockhaus firm in *Isis, oder enzyklopädische Zeitung*. The state of Sachsen-Weimar forced Oken to choose between his professorship or editing *Isis* in 1819. All journals published by Brockhaus were dedicated to the support of freedom of the press. Not surprisingly, the Austrian state in the Metternich era banned these journals, 1819–1823.

32 The uncensored version of André’s *National Calendar* (1821) is an extremely rare book. We examined one copy (104.894-B1821) stored in the Department for Manuscripts and Rare Books, Austrian National Library (Vienna) alongside three censored versions. André invested 80,000 Gulden of his own wealth in the publication of the *National Calendar* (Kucher 2007, p. 40). Due to censorship, the successful publication failed and André was effectively bankrupt.

33 André, to keep his word, had tried to edit ONV and publish the works sent to him. He was proceeding slowly, however, not only due to the ongoing efforts of the secret police, but also due to age-related issues, like his poor eyesight, and later the loss of his son Rudolf, who had been helping him with the editing. The task of editing ONV was later taken up by André’s younger son Emil André (1790–1869).

34 The letters of support sent to Hugo Salm can be found in the Moravian Archive (MZA) in Brno, G150, box 73, pp. 658–726. These letters, although difficult to read, are from seventeen members of the *Ackerbaugesellschaft* protesting against the removal of André. The following names or family names can be recognized from the handwriting: Freiherr Emanuel von Bartenstein, Count Colloredo, Eberl, J.M. Ehrenfels, Count Emmerich Festetics, Hening, Bricolini, Hochstetter, Schöftl (?), Herrn D. Ulram, Zimermann, Harvenfels (?), Lukiche (?), Count Prokop Lažanský, Ferenc Chernel, György Chernel, József Farkas.

35 Festetics’ name is spelled “Festeticz” in this reference. We found other spellings such as “Festetits” or “von Festetics,” which all refer to the same person and family.

36 Police reports of the Festetics family are listed under AT-OeStA/AVA Inneres PHSt 385, H66, PHSt 648, PHSt 2328, PHSt 106. None of these documents mention Imre Festetics, thus we assume he was not part of any investigation.

37 Documents numbered AT-OeStA/AVA Inneres PHSt 1820 (Kt 925/fol2-6). N.B., pages 3 to 6 were scorched and severely damaged.

38 E. Festetics, 1820b Letter to Count Hugo Salm. Kőszegpaty, 2 August 1820, Moravian Archive (MZA) in Brno, G150 box 80.

39 Caleb Hillier Parry literally wrote that inbreeding creates eunuch sheep (Parry 1806, p. 471).

40 Police report detailing André’s activities include AT-OeSTA/AVA Inneres PHSt 11,064 (1820); PHSt 9239 (1823); PHSt 1237, 2541, 67, 2118, H1 and H79. See further references listed in Píša (2018 p. 149).

41 Knight carried out hybridization experiments and published his results in 1799. He fertilized white peas with the pollen of grey (dominant) seeded peas and noted that the first-generation of hybrid plants had grey seeds. After backcrossing with the white (recessive) parent, he observed that the white type reappeared. He did not make any counts. Similar observations were made on peas in papers read to the members of the Horticultural Society in London by Alexander Seton on 20 August 1822, and by John Gross on 15 October 1822, while Knight presented his comments on these works on 3 June 1823. The papers of Seton, Gross, and Knight appeared in the same volume of the *Transactions of the Horticultural Society of London* in 1824 describing dominance and hybrid segregation.

42 Festetics is not specific about what traits he has observed.

43 Letter without any place or date, written and signed by Imre Festetics found in the Moravian Archive (MZA), Brno, G150 box 80. The handwriting and the inclination of the dip pen also assures that it was written by Festetics. It seems that the nib of the pen was rarely changed, resulting in a well recognizable but hard to read pattern, typical of Festetics’s letters sent from Paty and Kőszeg around 1820–1821. The letter was addressed to an “Honorable Count” whose identity remained hidden from us. Count Raitz in Festetics’s letter presumably refers to Hugo Salm, who was in contact with André and forwarding letters to him in Stuttgart.

44 Salm’s activities are listed in the police reports AT-OeStA/AVA Inneres PHSt 2031, PHSt 9445, while the correspondence is found in PHSt 6556. Cotta also published André’s journal *Hesperus* when he moved to Stuttgart until his death in 1831.

45 The Kingdom of Illyria was a crown land of the Habsburg Empire. It existed between 1816–1849, with Ljubljana (now Slovenia) as its administrative capital.

46 Documents numbered AT-OeStA/AVA Inneres PHSt 1826, Akt.2118/Kt.1147 from fol. 1 to 4, Austrian State Archives, Vienna. According to the surveillance report, the investigator became aware of a certain person named Martens, whose brother lives in Stuttgart, but he is not involved with editing. There are two pastors who moved from the district to Württemberg and could be potential mediators, but the most likely person is the canvas trader “Sigmund” who is in contact with a certain Konrad, who used to be an employee of the Prague City Council, now employed as a “*J.Ö. Appellationssekreter*.” Konrad carries out editing and printing for profit and, in January 1826, André was already in contact with Konrad, who included several articles by André published in Moravia. N.B., a few articles were mentioned but they are unrecognizable, and the text is scorched. Further recognizable parts state that the articles written by André might be identified by the context of foreign regulations (*Maßregeln*).

47 Naturally the Austrian government responded by banning *Hesperus*, and the chief of the political police ordered “intensive and extensive investigations” throughout the Monarchy among the subscribers of the journal. See the police reports about the incident in the Austrian State Archives under the numbers: AT-OEStA/AVA Inneres PHSt 2217, PHSt H122, and PHSt 4342/86qu. See the English translation of André’s *Price of Hundred Ducats* in the supplement of Poczai et al. (2021) and the description of Střesková (1966) and Kallbrunner (1925) about the incident.

48 The main data of Napp’s curriculum vitae were described by d’Elvert (1870, pp. 328–331), and it was explained in more detail by Weiling (1968). Like Mendel, Napp found opportunity for further studies in 1810 at the Augustinian monastery in Brno. In 1815, the gifted and talented Napp studied theology, but his interests included other subjects as well. At that time, he was entrusted with lectures at the Theological Institute in Brno and later concentrated on the field of linguistics. On 11 March 1824, he was elected abbot of the monastery and remained in this office until his death in 1867.

49 As the newly appointed abbot of the Augustinian monastery in Brno, Napp also became responsible for the management of the monastic estates. He quickly discovered that *lana potestas est*—the estates' economy are heavily reliant on the textile industry and sheep wool. Manufacturers and merchants had every reason to respect high-quality wool and support any method of acquiring it. The monastery's wealth was based on locally bred sheep, as well as the estate's development of novel varieties of fruits and vines, field crops, and ornamental plants.

50 The Cardinal Archbishop of Olomouc, Rudolf Johannes Joseph Rainier von Habsburg-Lothringen (1788–1831) was the brother of Francis II.

51 Moravian Archive (MZA) in Brno, Czechia, Archivestand 13, Sign. P113/8.

52 Nestler identified the animal and plant characteristics that determine agricultural output, as well as the *Anlage* of traits, but he did not separate the factors that control heredity; instead, he believed in blending inheritance. He also referred to the unexpected emergence of “deviations" (*Abweichungen*) as "freaks of nature" (*Naturspiel* or *Natursprung*). He gave the example of hornless cattle, which usually had hornless progeny, but on rare occasions, horned offspring appeared. He also attempted to distinguish between the impact of the environment and that of heredity, but described the inheritance of acquired traits as problematic.

53 The meaning of *Entwicklungsgeschichte* ascribed by Nestler obviously refers to the ontogenetic development of domestic varieties or breeds.

54 In 1827, the Cardinal Archbishop of Olomouc persuaded the emperor to promote the local Lyceum to university status, which took the name Francis University, with Faculties of Philosophy, Theology, and Law and a School of Medicine and Surgery. The Philosophical Institute's goal was to ease the transition from Gymnasium to the Theological Institute by providing two-year studies in "philosophy," which encompassed mathematics, physics, Greek, Latin, religion, history, while agriculture and natural sciences were compulsory (Klein and Klein 2013, pp. 256–252). Diebl applied for the latter professorship.

55 Ehrenfels specifically refers to Haller’s preface to the German translation of the second volume of Buffon’s *Histoire naturelle*, where he is raising critical questions about Buffon’s ideas on embryological development (1829, p. 130). These questions are focused on establishing what types of forces are responsible for development in the organization and production of the new organism (see Roe 1975, p. 176).

56 Mendel’s professor Franz Unger also applies the term; see Gliboff (1998) and Shan (2021).

57 For further details of this debate see Wood and Orel (2005, pp. 255–263).

58 Stieber also considered the transmission of traits from parents to offspring, but he found no correlations or patterns. Later, he also came into contact with Mendel and hinted that the organic action of the inner force (*innere Kraft*) deviates from its natural course due to external causes and such deviation has its limits because “it is not random but determined by certain laws of Nature” (Stieber 1842, p. 44). He saw heredity as an organic force and later tried to explain embryonic development in his book: “The power of life in generation fluids is so concentrated that even a little amount may create a new organism” (Stieber 1851, p. 123). Stieber also described sheep-goat hybrids and mentioned terms like, genetic identity (*genetische Identität*) (1862a, p. 52), genetic differences (*die genetische Unterschiede*) (1862a, p. 52) and genetic heredity (*genetische Vererbung*) (1862b, p. 94). Stieber also used the term *Befruchtungs-Elemente*, which recall Mendel’s *Elemente der Befruchtung* (Mendel 1866, p. 41).

59 Influential members are often referred to as „St.Thomas Quartet” composed of Gregor Mendel, Pavel Křížkovský (1820–1885), František Tomáš Bratránek (1815–1884), and František Matouš Klácel (1808–1882) (see Klein and Klein 2013, pp. 223–232).

60 Mendel's article in 1866 represented this novel methodology. The word *form* was being phased out in favour of *Gestalt* and *Beschaffenheit* (shape and consistency).

61 Chemistry was on the verge of becoming a self-contained natural science. Its status as a science assisting physics has been overcome via advancements in laboratory experimental activity. Much of physicists' experimental evidence was used in practice.

62 See the most recent translation of Mendel’s work by Müller-Wille and Hall (2020).
